# Reinvestigating an enigmatic Late Cretaceous monocot: morphology, taxonomy, and biogeography of *Viracarpon*

**DOI:** 10.7717/peerj.4580

**Published:** 2018-04-06

**Authors:** Kelly K.S. Matsunaga, Selena Y. Smith, Steven R. Manchester, Dashrath Kapgate, Deepak Ramteke, Amin Garbout, Herminso Villarraga-Gómez

**Affiliations:** 1 Earth and Environmental Sciences, University of Michigan, Ann Arbor, MI, USA; 2 Florida Museum of Natural History, University of Florida, Gainesville, FL, USA; 3 Department of Botany, Jashbhai Maganbhai Patel College, Bhandara, Maharashtra, India; 4 Imaging and Analysis Centre, Natural History Museum London, London, UK; 5 Nikon Metrology, Inc., Brighton, MI, USA

**Keywords:** *Viracarpon*, Plant, Fossil, Monocot, India, X-ray microCT, Late Cretaceous, Paleogene, Angiosperm

## Abstract

Angiosperm-dominated floras of the Late Cretaceous are essential for understanding the evolutionary, ecological, and geographic radiation of flowering plants. The Late Cretaceous–early Paleogene Deccan Intertrappean Beds of India contain angiosperm-dominated plant fossil assemblages known from multiple localities in central India. Numerous monocots have been documented from these assemblages, providing a window into an important but poorly understood time in their diversification. One component of the Deccan monocot diversity is the genus *Viracarpon*, known from anatomically preserved infructescences. *Viracarpon* was first collected over a century ago and has been the subject of numerous studies. However, resolution of its three-dimensional (3D) morphology and anatomy, as well as its taxonomic affinities, has remained elusive. In this study we investigated the morphology and taxonomy of genus *Viracarpon*, combining traditional paleobotanical techniques and X-ray micro-computed tomography (μCT). Re-examination of type and figured specimens, 3D reconstructions of fruits, and characterization of structures in multiple planes of section using μCT data allowed us to resolve conflicting interpretations of fruit morphology and identify additional characters useful in refining potential taxonomic affinities. Among the four *Viracarpon* species previously recognized, we consider two to be valid (*Viracarpon* *hexaspermum* and *Viracarpon* *elongatum*), and the other two to be synonyms of these. Furthermore, we found that permineralized infructescences of *Coahuilocarpon phytolaccoides* from the late Campanian of Mexico correspond closely in morphology to *V. hexaspermum*. We argue that *Viracarpon* and *Coahuilocarpon* are congeneric and provide the new combination, *Viracarpon phytolaccoides* (Cevallos-Ferriz, Estrada-Ruiz & Perez-Hernandez) Matsunaga, S.Y. Smith, & Manchester comb. nov. The significant geographic disjunction between these two occurrences indicates that the genus *Viracarpon* was widespread and may be present in other Late Cretaceous assemblages. *Viracarpon* exhibits character combinations not present in any extant taxa and its affinities remain unresolved, possibly representing an extinct member of Alismatales. The character mosaic observed in *Viracarpon* and the broad distribution of the genus provide new data relevant to understanding early monocot evolution and suggest that the (thus far) largely invisible Late Cretaceous monocot diversification was characterized by enigmatic and/or stem taxa.

## Introduction

During the Cretaceous period (∼145–66 Ma) angiosperms (flowering plants) underwent a rapid evolutionary radiation, first appearing in the fossil record around 140 million years ago and rising to geographic and ecological dominance in terrestrial ecosystems by the end of the Cretaceous (McElwain, Willis & Lupia, 2005; Berendse & Scheffer, 2009; Herendeen et al., 2017). The origin and diversification of angiosperms has been studied intensively by evolutionary biologists (Crane, Friis & Pedersen, 1995) but many questions on the timing of evolutionary events and relationships between lineages remain unresolved, particularly for monocots, which comprise approximately one-fifth of extant angiosperm species (Friis, Pedersen & Crane, 2004). The fossil record is especially important for addressing these questions because fossils provide minimum ages for lineages, empirical data on past diversity, and evidence of character combinations not present in extant taxa (Crepet, Nixon & Gandolfo, 2004; Smith, 2013). Pollen and macrofossils indicate an Early Cretaceous origin for monocots and subsequent diversification during the Late Cretaceous (Friis, Pedersen & Crane, 2004; Crepet, 2008; Smith, 2013). However, owing to their sparse fossil record during this time, details of the Late Cretaceous diversification of monocots are poorly understood (Crepet, 2008) and new discoveries are essential for shedding light on the tempo and mode of monocot evolution.

The fossil floras of the Maastrichtian–Danian (∼72–61 Ma) Deccan Intertrappean Beds of India are an important source of data for understanding angiosperm evolution and historical biogeography. Hundreds angiosperm taxa have been documented, roughly half of which are monocots (Kapgate, 2009; Srivastava, 2011; Wheeler et al., 2017), providing a window into monocot diversity during the Late Cretaceous when India was geographically isolated in the southern hemisphere tropics (Ali & Aitchison, 2008; Chatterjee, Goswami & Scotese, 2013). Moreover, connections with Gondwanan landmasses during the Jurassic, millions of years of geographic isolation following continental breakup, and subsequent physical connection with Eurasia for most of the Cenozoic raise the possibility that India had an important role in the biogeographic history of plant groups. Possible scenarios include high endemism stemming from geographic isolation, or “out-of-India” dispersal of Gondwanan lineages into Eurasia (Morley, 2003; Karanth, 2006; Whatley & Bajpai, 2006; Manchester, Kapgate & Wen, 2013). The excellent preservation of many of these permineralized fossils facilitates resolution of key morphological characters and makes precise taxonomic identifications possible (Manchester, Kapgate & Wen, 2013; Manchester et al., 2016), which are necessary for answering broader questions of evolution and biogeography. However, despite over a century of studies on the flora some of the taxa have eluded attempts to determine precise systematic relationships. One of these enigmatic plants is *Viracarpon*, a monocot found abundantly in the Deccan Intertrappean Beds.

*Viracarpon* comprises compact infructescences bearing sessile, six-seeded fruits, known from at least eight separate localities of the Deccan Intertrappean Beds (Kapgate, 2009), as well as in underlying sediments of the Lameta Formation (Samant & Mohabey, 2009). “Mulberry-like fruits” were first mentioned by Hislop (1853) and the fossils were later formally described as a new monocot genus, *Viracarpon*, by Sahni (1934). Four species have been named: *Viracarpon hexaspermum* Sahni (1934), *Viracarpon elongatum* Sahni (1944), *Viracarpon sahnii* Chitaley, Shallom & Mehta (1969), and *Viracarpon chitaleyi* Patil (1972). The presence of scattered vascular bundles in the peduncle and trimerous floral architecture indicate affinities with monocotyledons, and relationships with the Araceae, Pandanaceae, Cyclanthaceae, and Scheuchzeriaceae have all been suggested (Sahni, 1944; Chitaley, 1954; Verma, 1958; Nambudiri & Tidwell, 1978). However, the complex morphology of the fruits and unusual combination of characters have led to considerable disagreement over synonymy of species, interpretation of morphology, and the familial placement of the genus. Moreover, the unusual morphology of *Viracarpon* and lack of clear taxonomic affinities suggest that it may provide an example of a genus that was endemic to India, reflecting the prolonged isolation of the subcontinent.

We reinvestigated *Viracarpon* using light microscopy and X-ray micro-computed tomography (μCT) to reconstruct the complex morphology of fruits and infructescences. The reconstructions help to resolve conflicting interpretations among earlier researchers and reveal new characters necessary for determining systematic affinities. Based on these results, we provide here a revised taxonomy and emended descriptions of the species. Further, the hypothesis that *Viracarpon* is endemic to India is rejected based on our reevaluation of *Coahuilocarpon phytolaccoides* infructescences from the late Campanian of Mexico. *Viracarpon* provides new data on the Cretaceous radiation of monocots and its reevaluation represents an important step toward using these data to address broader questions on angiosperm evolution.

## Materials and Methods

The Deccan volcanic province (DVP) comprises a sequence of continental flood basalts (traps) formed during the late Maastrichtian to early Danian (67–64 Ma, chrons 30N–29N; Hooper, Widdowson & Kelley, 2010; Renne et al., 2015; Schoene et al., 2015), which crops out across central and western peninsular India. Intertrappean sedimentary layers, deposited during quiescent intervals between volcanic eruptions, contain abundant three-dimensionally preserved plants with cellular details documented from approximately 57 different exposures (Kapgate, 2009). Although the precise ages of many fossil localities are poorly constrained, most can be broadly considered either late Maastrichtian or early Danian depending on their location within the DVP and stratigraphic continuity with dated outcrops (Srivastava & Guleria, 2006; Smith et al., 2016).

Specimens of *Viracarpon* have been recovered from eight different localities: Mohgaonkalan, Keria, Phutala Tank, Mahurzari, Dongargaon, Takli, Singpur, and Marai Patan (Sahni, 1934; Chitaley, Shallom & Mehta, 1969; Nambudiri & Tidwell, 1978; Kapgate, 2009; this study). Within the state of Maharashtra, the Phutala Tank, Mahurzari, and Takli localities are part of the Nagpur district, while Dongargaon and Marai Patan are in the Chandrapur district. These localities are within the northeastern edge of the Deccan main plateau. Mohgaonkalan, Keria, and Singpur are in the Chhindwara district of Madhya Pradesh, and are in the southwestern portion of the Mandla lobe of the DVP. Most localities are found in or around small villages for which they are named, and more precise directions and descriptions are given by Kapgate (2009). The intertrappean beds at Takli, the type locality for *Viracarpon*, were located within the city of Nagpur but have since been completely excavated by building construction and are no longer accessible.

Chronostratigraphic investigations of the Phutala Tank, Mahurzari, Dongargaon, Takli, and Marai Patan localities have not been conducted to determine their age as either late Maastrichtian or early Danian. However, the presence of palynofloras containing Maastrichtian indicator taxa at the Singpur locality (Samant, Mohabey & Kapgate, 2008), as well as Maastrichtian vertebrates at the Naskal locality (ca. 100 km southwest of Nagpur; Rage, Prasad & Bajpai, 2004), suggest that a late Maastrichtian age for these localities is likely. The Mohgaonkalan locality is considered late Maastrichtian in age, based on evidence from palynology, vertebrate fossils, and magnetostratigraphy indicating deposition during chron 30N (Samant & Mohabey, 2009). Keria is part of the same intertrappean as Mohgaonkalan, and is also considered late Maastrichtian (B. Samant, 2017, personal communication). All current information on the age of the paleobotanical localities indicate that the Indian *Viracarpon* fossils are most likely late Maastrichtian in age.

Specimens of *C. phytolaccoides* were loaned by Dr. Sergio Cevallos-Ferriz (Universidad Nacional Autónoma de México (UNAM)). They were originally collected from the Cerro del Pueblo Formation in the southwestern region of the state of Coahuila in Mexico (Cevallos-Ferriz, Estrada-Ruiz & Perez-Hernandez, 2008). The Cerro del Pueblo Formation is late Campanian in age (72.3–71.3 Ma) based on ammonite biostratigraphy and magnetostratigraphy (Kirkland et al., 2000; Cevallos-Ferriz, Estrada-Ruiz & Perez-Hernandez, 2008). Specimens are curated at the Colección Nacional de Paleontología, Instituto de Geología, UNAM, in Mexico City, Mexico.

*Viracarpon* specimens examined in this study included petrographic thin sections, peels (cellulose acetate or butyl acetate), and intact specimens curated at the Florida Museum of Natural History (FLMNH), University of Kansas Biodiversity Institute (KU), Cleveland Museum of Natural History (CMNH), Natural History Museum London (NHMUK), and the Birbal Sahni Institute of Paleobotany (BSIP) in Lucknow, India. Figured specimens include those accessioned at NHMUK (V35059, V35038, V35036), CMNH (P24742, P3771, slide 4305), FLMNH (UF19442-70248), and BSIP (9349, 9351). NHMUK specimens originate from the Takli locality. Figured specimens from BSIP and CMNH were collected from Mohgaonkalan. One specimen of *V. elongatum* (UF19442-70248) was collected from the Marai Patan locality. *Viracarpon* fossils comprise whole infructescences permineralized in chert and preserved to the tissue and sometimes cellular level, with little or no compression of organs. The completeness of specimens and preservation of fine structures such as spines and trichomes indicates that most infructescences experienced relatively little transport prior to fossilization. Specimens of *Coahuilocarpon* comprise whole infructescences preserved by iron silicate permineralization (Rodríguez-de la Rosa & Cevallos-Ferriz, 1994), and exhibit varying degrees of compression. However, the specimen figured here is more or less uncompressed.

In order to accurately reconstruct the three-dimensional (3D) morphology of *Viracarpon* and non-destructively study valuable museum specimens, many of the fossils were studied using μCT (Smith et al., 2009; Collinson et al., 2012, 2016). In a μCT scan, X-rays are emitted by a microfocus X-ray tube and pass through a rotating sample to an X-ray detector, which takes a series of two-dimensional (2D) radiographic projection images as the sample rotates. Denser materials absorb more of the X-ray photons, allowing fewer of them to reach the detector. In reconstructed μCT scans, for which the internal structural information has been computed from the projection images, denser materials are represented by lighter gray values while less dense ones are darker. All figured specimens, except those from NHMUK, were scanned at Nikon Metrology (Brighton, MI, USA) and the University of Michigan CTEES facility with a Nikon XT H 225ST industrial μCT system using a tungsten reflection target. Specimens curated at NHMUK were scanned using a Nikon HMX ST225 μCT system, at the NHMUK Imaging and Analysis Centre. Depending on the specimen, scans were set at 100–180 kV, 120–300 μA, and used 0–2 mm of copper filter, which reduces strong artifacts in reconstructed images by suppressing lower energy X-rays. Pixel size varied from ca. 14–20 μm. All scans were acquired using Inspect-X and reconstructed using CT Pro 3D (Nikon Metrology, USA), which uses a FDK (Feldkamp–Davis–Kress) type algorithm. This software takes the 2D projection images acquired by the X-ray detector and generates a 3D image represented by gray values that are distributed in a volumetric space. Reconstructed datasets were analyzed and 3D models were produced using Avizo 9 Lite 3D software (FEI, Hillsboro, OR, USA). We refer to sections obtained from the reconstructed μCT data as “digital sections.” Volume renderings were produced using both the “volren” and “volume rendering” modules in Avizo, while 3D models were done by manual segmentation of μCT scans. 3D models were converted to U3D format using MeshLab (http://meshlab.sourceforge.net/) for generating 3D PDF files using Adobe Acrobat Pro IX (Adobe, San Jose, CA, USA; Fig. S1). Figures were constructed using CorelPhoto-Paint X8 and CorelDraw X8, and supplemental videos produced using Corel VideoStudio X10 (Corel Corporation, Ottawa, ON, Canada).

Raw μCT data (projection files, volumes, and image stacks) for figured specimens curated at NHMUK are archived by the museum Imaging and Analysis Centre (IAC). Archived data can be accessed by contacting staff at IAC (http://www.nhm.ac.uk/our-science/departments-and-staff/core-research-labs/imaging-and-analysis-centre.html). Additional video files of figured specimens V35059 and V35036 are available in the Supplemental Information (Videos S3–S6). Raw μCT data (tiff stacks) for figured CMNH, FLMNH, and UNAM specimens are available publicly on MorphoSource (Duke University; https://www.morphosource.org/Detail/ProjectDetail/Show/project_id/430) under project number P430 (title: Viracarpon, an enigmatic monocot from the Late Cretaceous). Additional video files and surfaces (.ply format) for these specimens are also available in the MorphoSource project.

## Results

**Systematic paleobotany**
**Division**—**Magnoliophyta**
**Class**—**Liliopsida**
**Family**—**incertae sedis**
**Genus**—***Viracarpon*** Sahni, Proc. 21st Ind. Sci. Cong. 21: 318. 1934.

*Emended generic diagnosis*—Infructescence compact, subglobose to elongate; fruits densely inserted, sessile, arranged helically or in whorls; peduncles slender, with scattered vascular bundles; gynoecia composed of six single-seeded carpels, semicarpous; ovaries fused centrally, lateral walls of adjacent carpels free or partially fused, styles free; each ovary with prominent central axis; six vascular bundles per fruit in central axis, each bundle opposite of and extending over top of locule distally; placentation apical, each seed vascularized by single bundle entering from distal end of locule.

**Figure 1:**
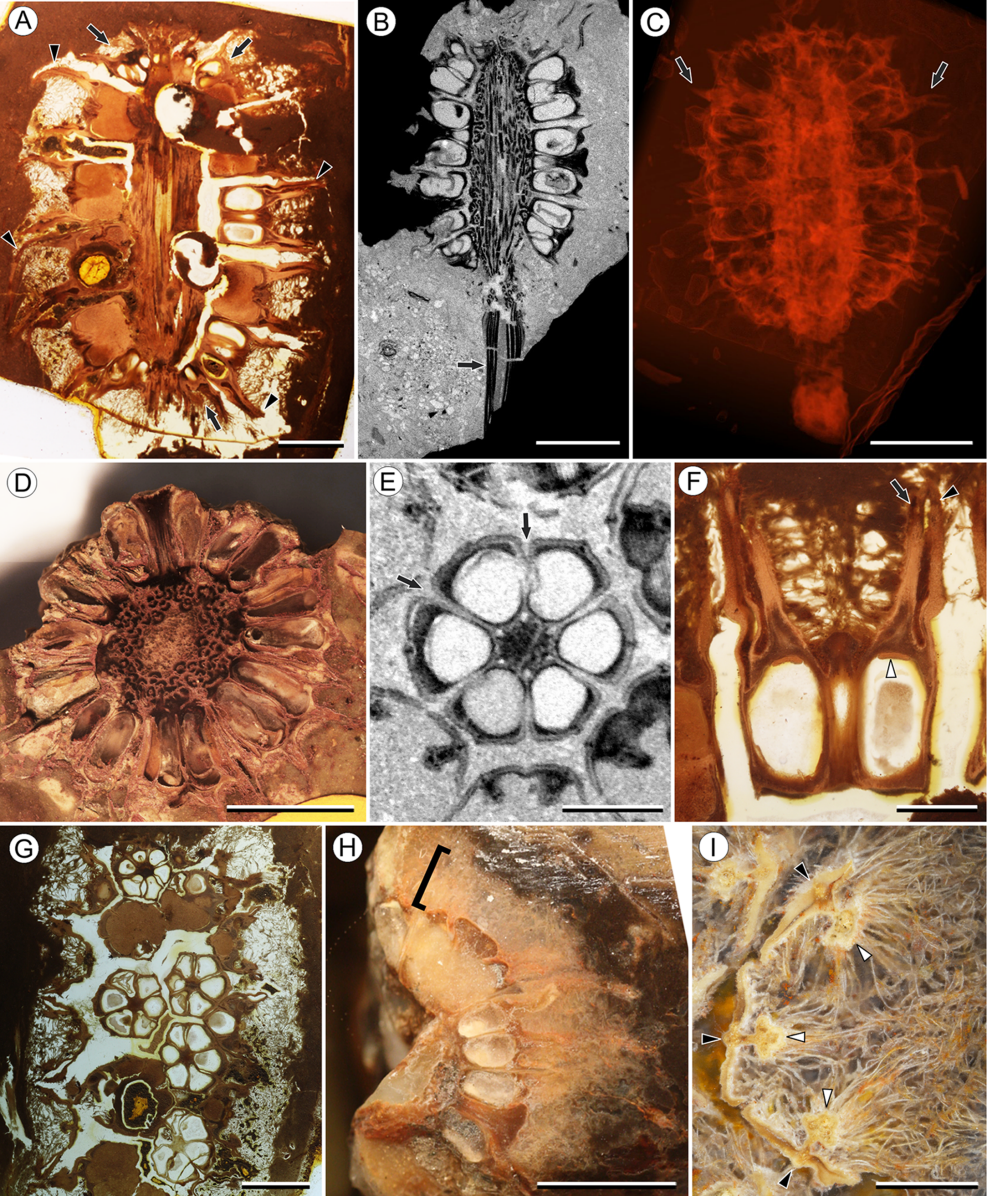
Infructescence and fruit structure of *Viracarpon hexaspermum* Sahni emend. Matsunaga, S.Y. Smith, & Manchester. (A) Light micrograph of longitudinal section (LS) through infructescence. Note the compact organization of sessile fruits, and the spines extending from the distal end of each fruit (black arrowheads). Reduced fruits at apical and basal regions of infructescence are indicated by arrows. Scale = 5 mm. BSIP 9349. (B) Digital LS of infructescence. Note vascular bundles seen in LS (arrow), and that the diameter of the peduncle is much wider where fruits are attached. Scale = 5 mm. NHMUK V35059. (C) Volume rendering of μCT data of specimen shown in (B). Note spines extending from fruits (arrows). Scale = 5 mm. NHMUK V35059. (D) Transverse section (TS) through infructescence. Note dense organization of fruits and scattered arrangement of vascular bundles in peduncle. This image is a composite of two detail images, stitched together using Photoshop CC. Scale = 5 mm. NHMUK V35038, holotype. (E) Digital TS through fruit showing central axis around which carpels are organized, and gaps between adjacent carpels (arrows) indicating only partial fusion. Note the perianth and styles of adjacent fruits, intercepted in more distal planes of section. Scale = 2 mm. NHMUK V35059. (F) LS through fruit showing projecting styles (arrow) and perianth (black arrowhead). Note trichomes on spines and perianth, and lenticular hypostase-like tissue at distal end of locule (white arrowhead). Scale = 2 mm. BSIP 9349. (G) Tangential section through infructescence showing helical arrangement of fruits producing clear orthostichies. Scale = 5 mm. BSIP 9351. (H) TS through infructescence showing the dense trichomes that characterize some specimens (trichome halo indicated by the bracket). Scale = 5 mm. CMNH P24742. (I) TS through three perianth elements (black arrowheads) and attached styles (white arrowheads), showing trichomes on the adaxial surface of both structures. Individual trichomes can be relatively long, filling space above the fruit. Scale = 2 mm. CMNH P24742.

**Figure 2:**
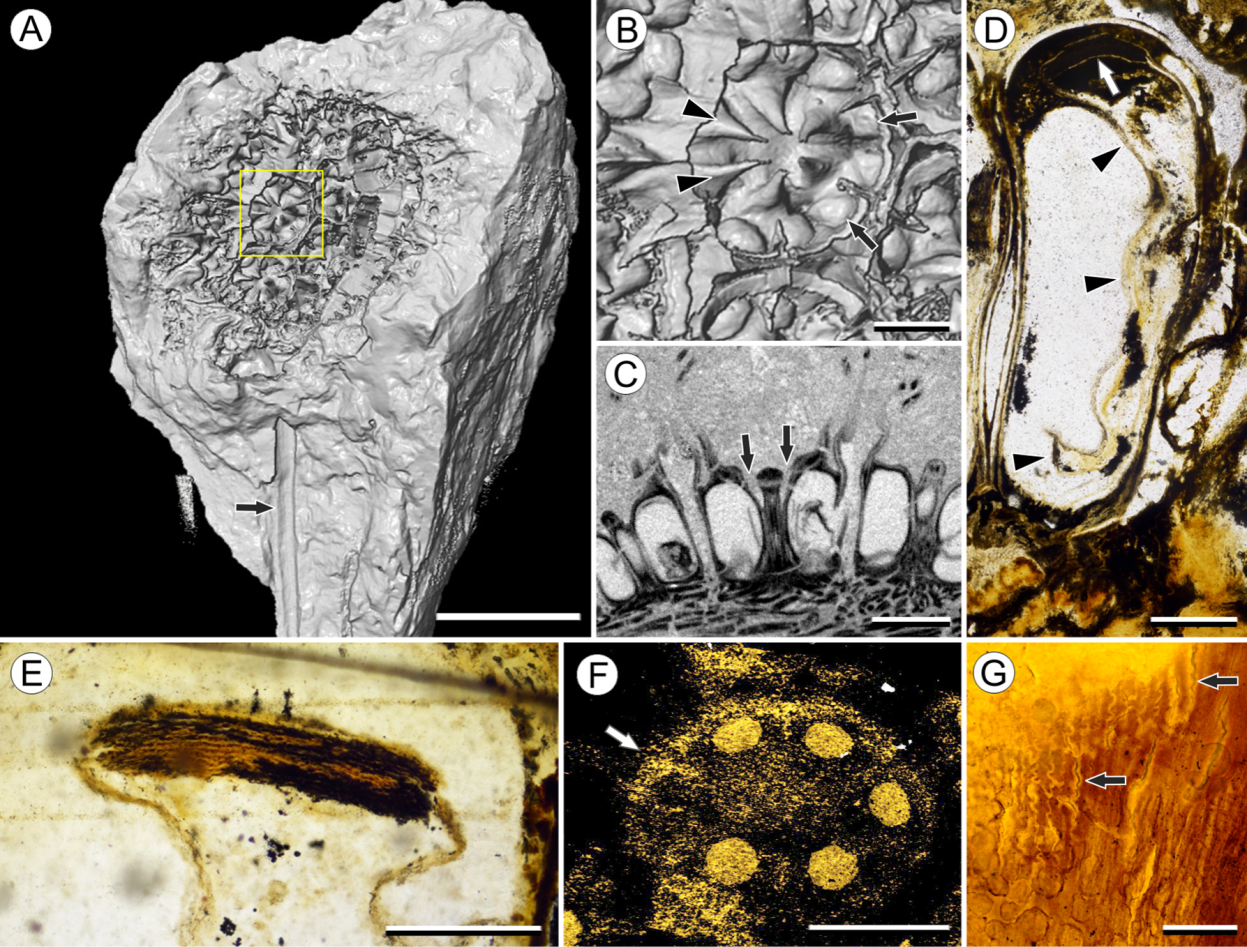
Fruit and seed structure of *Viracarpon hexaspermum* Sahni emend. Matsunaga, S.Y. Smith, & Manchester. (A) Volume rendering of an infructescence exhibiting an unusual mode of preservation: perianth and spines are preserved in rock matrix, but remainder of infructescence has eroded away leaving behind a mold of its external surface and revealing structural detail otherwise difficult to see, such as the six channels and 12 depressions on fruit surfaces. Note impression of slender peduncle (arrow), and nearly globose shape of this particular infructescence. Detail image of boxed area is shown in (B). Scale = 10 mm. NHMUK V35036. (B) Detail of individual fruit showing the 12 depressions (arrows), and molds of the six channels that extend from upper surface of each fruit (arrowheads). Scale = 2 mm. NHMUK V35036. (C) Digital longitudinal section (LS) of a fruit where plane of section passes through two of the channels (arrows) shown as molds in (B). Scale = 2 mm. NHMUK V35059. (D) LS light micrograph of peel section showing a seed with seed coat (arrowheads) pulled away from left side of locule wall. Attached at distal end of the seed is a lenticular, hypostase-like tissue (arrow). Scale = 500 μm. NHMUK V35038. (E) LS light micrograph of peel section showing detail of hypostase-like cap inside of the seed. Note structure is positioned inside seed coat. Scale = 500 μm. NHMUK V35038. (F) Volume rendering of fruit showing 3D view of hypostase-like structures (circular shapes) shown in (D) and (E). Note that volume renderings can be adjusted to display regions of μCT image histograms that correspond to density features. Because the hypostase-like caps are a different density than other parts of the fruit, in this image only the caps within the locules are clearly visible, but the faint outline of the fruit can be seen (arrow). Scale = 2 mm. CMNH P24742. (G) Light micrograph of peel section through pericarp. The locular epidermis is characterized by elongate cells with sinuous walls (arrow). Note the narrower, elongate cells (bottom right) and isodiametric cells (bottom left) corresponding to other pericarp tissues. Scale = 200 μm. CMNH slide 1754.

**Figure 3:**
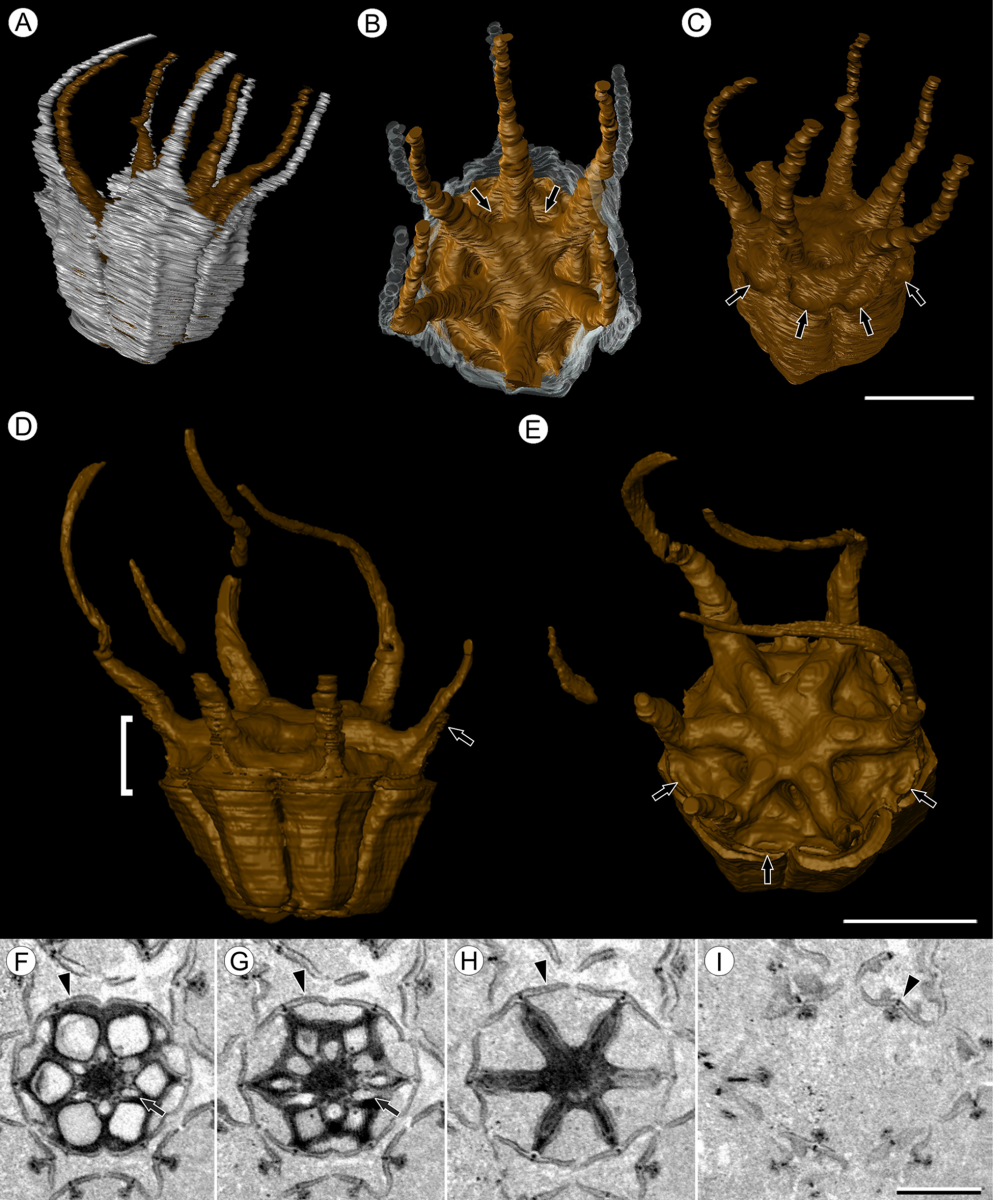
Fruit morphology of *Viracarpon hexaspermum* Sahni emend. Matsunaga, S.Y. Smith, & Manchester. (A–E) 3D models of fruits from segmented μCT data. (A) Side view showing tapering of perianth (white) into spine-like structures and parallel orientation of style and perianth spines. Note that spines continue for much greater lengths than shown, but were difficult to accurately segment beyond the point illustrated. (B) Upper surface showing stellate pattern formed by styles and six channels between the style bases (arrows). (C) Oblique lateral view with perianth removed to show four of 12 depressions on outer edge (arrows). (A–C) Scale = 2 mm. CMNH P24742. (D) Side view of fruit; perianth is not shown above where it separates from the fruit. Note grooves along fruit corresponding to unfused parts of the carpels, flanges that attach styles to perianth (arrow) illustrating the length along which the two organs are fused, and long and sinuous shape of the styles. Bracket indicates region shown in transverse sections (TS) in (F). (E) Upper surface of fruit in (D), showing same structures as seen in (B) but with higher resolution. Note six channels positioned between style bases and 12 depressions (arrows), which appear much shallower in this specimen than those shown in (C). (D and E) Scale = 2 mm. NHMUK V35059. (F–I) Serial digital TS through the bracketed region in (D). Note channels (arrows) that widen distally, stellate structure formed by styles (H), attached perianth elements (arrowheads), and perianth elements with attached styles in TS above level of ovary (I). Scale = 2 mm. NHMUK V35059. See also Fig. S1.

**Type Species**—***Viracarpon hexaspermum*** Sahni emend. Matsunaga, S.Y. Smith, & Manchester.
*Basionym: Viracarpon hexaspermum* Sahni, Proc. 21st Ind. Sci. Cong. 21: 318. 1934.
*Synonyms: Viracarpon sahnii* Chitaley, Shallom & Mehta, J. Sen Mem. Vol. Bot. Soc. Bengal: 333. 1969.
*Holotype*: V35038 (NHMUK; Fig. 1D), Sahni, 1944, Figs. 25–28.
*Other specimens studied*: V35059 (NHMUK; Figs. 1B, 1C, 1E, 2C and 3D–3I), V35056 (Figs. 2A and 2B), V35038 (Figs. 2D and 2E), CMNH P24742 (Figs. 1I, 1H, 2F and 3A–3C).
*Type locality, stratigraphy, and age*: Takli—Deccan Intertrappean Beds, India, Late Maastrichtian–early Danian.
*Other occurrences*: Mohgaonkalan, Mahurzari, Phutala Tank, Singpur, Marai Patan—Deccan Intertrappean Beds, India, late Maastrichtian–early Danian.

*Emended specific diagnosis*—Infructescences globose to ellipsoid, up to 3.0 cm long and 2.0 cm wide, fruits helically arranged; peduncle wider in region of fruit attachment, containing three to four cycles of vascular bundles; outer vascular bundles smaller than innermost ones; fruits (excluding styles and perianth elements) up to 5.0 mm wide and 5.0 mm long, size variable between specimens; lateral walls of adjacent carpels in ovaries free; styles persistent, spine-like, forming prominent ridges on distal surface of fruit, fused to perianth basally; six channels extending down from top of fruit between adjacent carpels; six inner perianth elements, persistent, fused to ovary and style bases, tapering into sinuous spine-like structures; adaxial surface of styles and perianth densely trichomatous; fruit vascularized by six bundles extending from the base of the fruit into the styles.

Description:

Infructescence structure: Infructescences of *V. hexaspermum* are generally ca. 1.0–2.0 cm wide and up to 3.0 cm tall (Figs. 1A–1D). The peduncle is slender, 3.0 mm wide, and is expanded apically to about 6.0 mm wide where fruits are attached (Figs. 1B and 1C). Fruits are sessile and exhibit a compact helical arrangement, with little or no space between adjacent fruits, forming ca. 8–12 orthostichies (Figs. 1A–1H). Reduced or abortive fruits are present near the base and at the very apex of infructescences (Fig. 1A, arrows).

Fruit structure: Most fruits range from about 4.0–6.0 mm wide and 5.0 mm tall, excluding the spines attached to each fruit (Figs. 1E and 1F). The gynoecia are semicarpous, composed of six single-seeded carpels fused centrally and forming a thick axis around which the locules are arranged (Figs. 1E and 1G). This central fusion extends the entire length of the ovary. Lateral walls between adjacent carpels are not fused but are often closely appressed (Figs. 1E and 1G). Seeds are usually poorly preserved, represented by a very thin seed coat with a lenticular cap of tissue on the distal end, which may represent a hypostase (Figs. 2D–2F). No convincing embryo or endosperm tissues have been observed, and seed coat preservation is too poor to determine thickness or histology. The pericarp is thin, mostly sclerenchymatous, and the fruits are densely packed in the infructescence, indicating they were probably not very fleshy at maturity. Detailed anatomical descriptions were given by Chitaley (1954), but the cellular preservation of specimens studied here is generally poor and it is difficult to confirm the histological observations made by previous researchers. However, in some specimens grazing sections through the pericarp show that the innermost cell layer, which lines the locule, is comprised of elongate cells with sinuous walls, approximately 40 μm wide and 200 μm long (Fig. 2G). This anatomy was also documented by Chitaley (1954), who also noted a layer of fibers to the inside of the locular epidermis. The distal end of the fruit possesses a sclerenchymatous tissue that forms a stellate pattern of ridges, which radiate from a central depression. Each ridge forming a spine that extends distally from the outer edge of the fruit (Figs. 3B–3E; Video S1). The position of the spines above each locule, as well as patterns of vascularization (described below) suggest that these spines are persistent styles. No dehiscence structures, germination sutures, or opercula have been observed.

**Figure 4:**
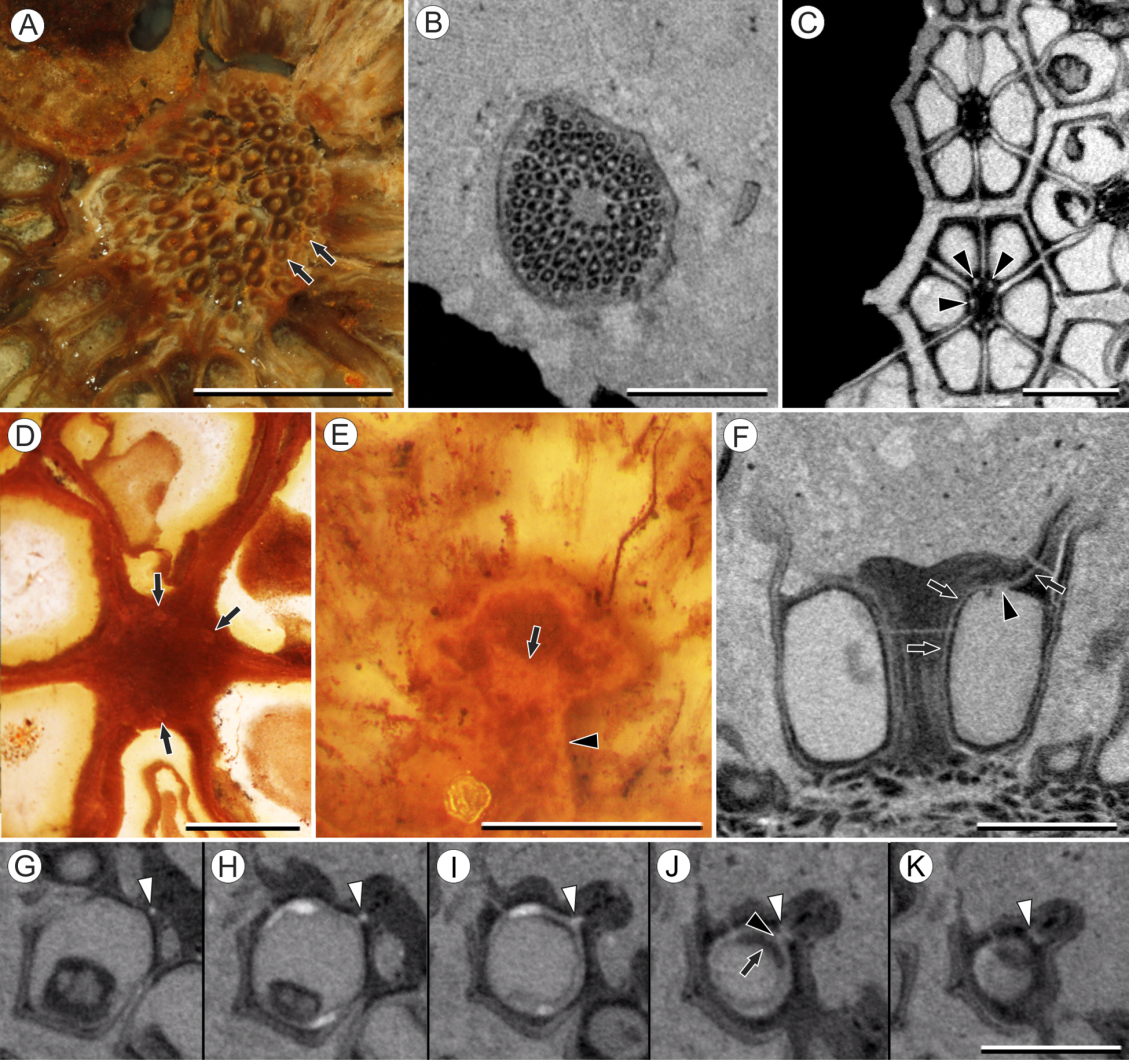
Vasculature of *Viracarpon hexaspermum* Sahni emend. Matsunaga, S.Y. Smith, & Manchester. (A) Light micrograph (LM) of thin section showing stele of peduncle from region of fruit attachment. Note scattered organization of bundles, fibrous sheaths that often completely surround conducting cells, and smaller size of outermost vascular bundles (arrows). Scale = 5 mm. CMNH P24742. (B) Digital transverse section (TS) through peduncle below zone of fruit attachment showing scattered organization of vascular bundles, narrow central pith, and decrease in bundle size toward outside of stem. Scale = 2 mm. NMHUK V35059. (C) Digital TS of fruits showing six vascular bundles in central axis (arrowheads). Scale = 2 mm. NHMUK V35059. (D) LM TS through central axis of a fruit showing the vascular bundles (arrows). Scale = 2 mm. BSIP 9351. (E) LM TS through style showing vascular bundle (arrow) and flange of tissue (arrowhead) that attaches it to perianth (not visible). Scale = 1 mm. BSIP 9351. (F) Digital longitudinal section of fruit showing trajectory of one vascular bundle (arrows). Near distal end of fruit the bundle branches, one branch entering the locule at a shallow angle through a gap in the tissues surrounding the locule (arrowhead), and the other branch continues up into the style. Note that the bundle appears as a lighter gray relative to the other fruit tissues. Scale = 2 mm. NHMUK V35059. (G–K) Serial digital TS through a fruit from top of locule, where vascular bundle diverges into it. (G) and (H) show vascular bundle (white arrowhead) close to locule. In (I), bundle crosses into locule through a gap in locule wall. (J) Bundle has bifurcated. One branch has diverged into locule wall, as seen in (J), and is visible between locule wall and seed coat (black arrowhead). The second branch (white arrowhead) will enter style. Note thickened zone of seed coat corresponding to hypostase-like tissue (arrow). (K) Branch of vascular bundle in base of style (white arrowhead). Note that the bundle is now wider than the original bundle seen in (G). Scale = 1 mm. NHMUK V35059.

Vasculature: Transverse sections through the peduncle reveal three to four cycles of scattered vascular bundles arranged around a narrow central pith (Figs. 4A and 4B). Vascular bundles tend to be larger toward the center of the stem both below and within the zone of fruit attachment, and thus the smaller peripheral bundles are not vascular traces supplying individual fruits (Figs. 4A and 4B). Preserved vascular bundles are composed fibers that either surround the vascular tissues or form two caps adjacent the xylem and phloem. Xylem tracheary elements are sometimes preserved (Chitaley, Shallom & Mehta, 1969), but phloem anatomy was not preserved in our specimens and has not been documented by other authors. Individual fruits are vascularized by six bundles, which run through the central axis of the fruit, positioned opposite the locules (Figs. 4C and 4D). In digital μCT sections these bundles appear white or light gray, contrasting with the darker and less dense organic material of the preserved plant tissues (Fig. 4C). This probably results from infilling of the conducting tissues with the dense siliceous matrix in which the fossils are preserved. Similarly, in the vascular bundles of the peduncle the fiber caps are well-preserved, but the conducting tissues are not and exhibit gray values similar to those of the matrix (Fig. 4B). Each bundle in the fruit vascularizes a seed as well as the corresponding spine. The bundle extends up through the central axis of the fruit and curves laterally over the top of the locule where it bifurcates, with one strand immediately entering the locule wall and the other continuing apically into the spine (Figs. 4E–4K). This is most easily seen in μCT videos through successive digital slices that follow the trajectory of the bundles through the fruit (Figs. 4G–4K; Video S2). The strand that continues into the spine is wider than the original bundle (Figs. 4E and 4K), but resolution in μCT scans is insufficient to ascertain whether it composed of a single strand or of multiple closely spaced strands. The consistent vascularization of each locule wall at the distal end indicates apical placentation of ovules. This is congruent with the apical attachment of seeds within the locules observed by Chitaley (1954). Although nearly all monocots with apical placentation have orthotropous ovules (Dahlgren, Clifford & Yeo, 1985), preservation is insufficient to confirm this ovule orientation.

Perianth: Each fruit has six perianth elements that are fused to both the ovary wall and the bases of the persistent styles (Fig. 3). Above the ovary, these perianth elements are laminar for several millimeters before tapering into long, slender, spine-like structures that run parallel with the persistent styles (Figs. 3A and 3F–3I). In many specimens both the persistent style and the adaxial surface of the perianth are covered densely in long, sinuous trichomes (Figs. 1H and 1I). The absence of trichomes in some specimens could reflect either differential degradation or intraspecific variation.

Other structures: Two other notable features of the fruit were observed. The first is the presence of six hollow, tapering channels in the fruits positioned between the bases of each style where they diverge from surface of the fruit (Figs. 3B–3F). These channels run approximately one-third of the way down the length of the fruit and are aligned with, but not connected with, the gaps between the partially fused carpels (Figs. 2A–2C and 3F). The second feature consists of 12 depressions along the perimeter of the distal surface of each fruit (Figs. 3C and 3E). Each style is flanked by two such depressions. It is unclear what these two features represent. The position of the six channels suggests they could be the remnants of a septal nectary, which are common in some monocots (Rudall, 2002), but no anatomical information to support this is preserved. They may alternatively be additional gaps between carpels, possibly formed during fruit maturation. The 12 depressions may be formed incidentally by the fusion of the style and perianth in that region, but it is alternatively possible that they represent the attachment points of stamens. Chitaley (1954) described vascular bundles in the pericarp below these depressions. However, no direct evidence of stamens or pollen have been observed in any specimens, and preservation of specimens studied here is insufficient to confirm whether the depressions are vascularized.

**Figure 5:**
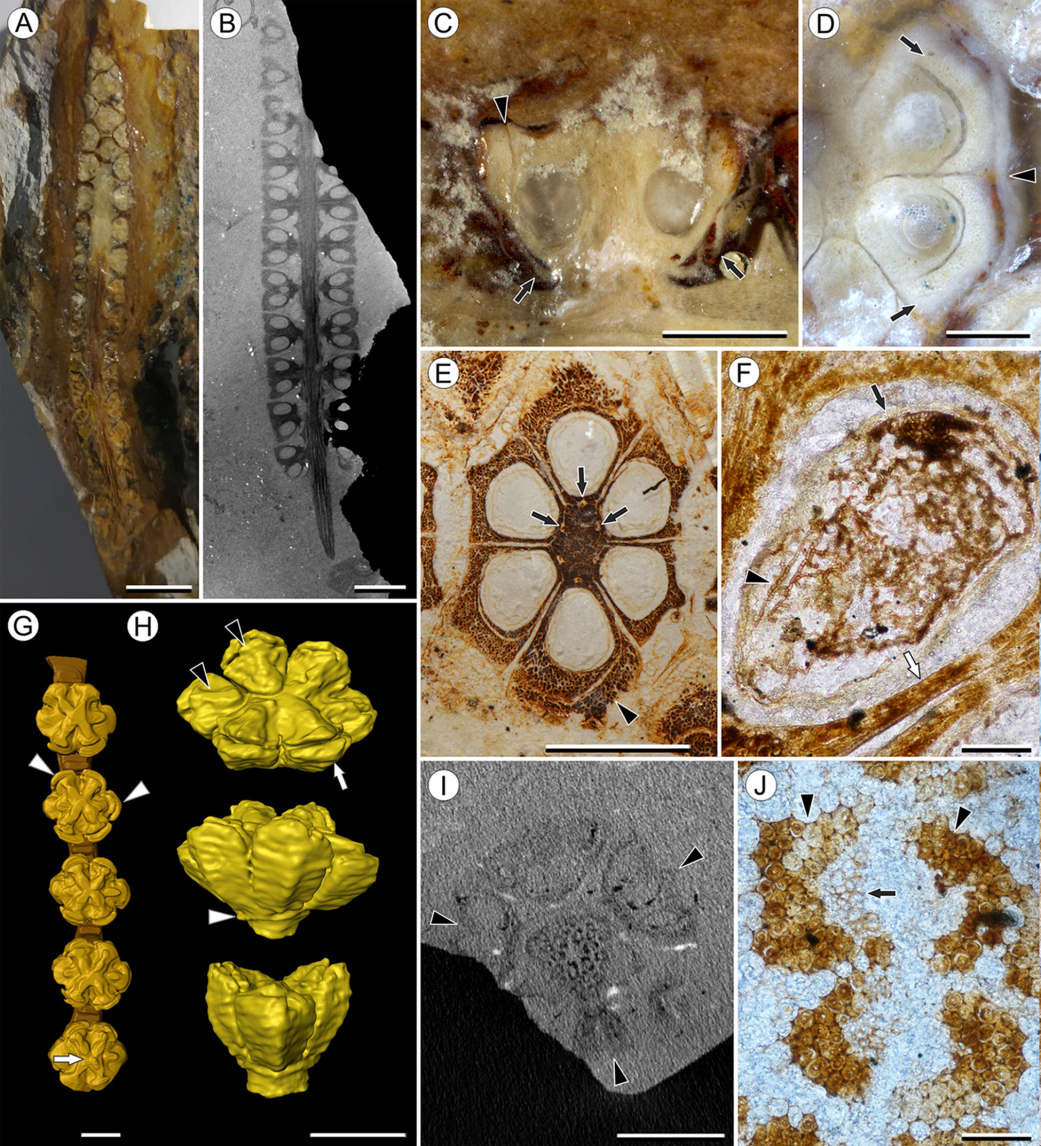
*Viracarpon elongatum* Sahni ex Chitaley & Patil emend. Matsunaga, S.Y. Smith, & Manchester. (A and B) Longitudinal sections (LS) through the infructescence seen exposed on surface of specimen (A) and in μCT scan (B). Note opposite arrangement of fruits (the third orthostichy in the whorl is not seen in this plane of section) and uniform width of peduncle below point of fruit attachment in (B). Scale = 5 mm (A), 2 mm (B). CMNH P3771 (A), UF19442-70248 (B). (C) Detail of fruit exposed in LS from specimen shown in (A). Note free lobes of outer perianth whorl (arrows) and gap delimiting free portion of inner perianth whorl (arrowhead). Scale = 1 mm. CMNH P3771. (D) Transverse section (TS) of fruit from specimen shown in (A). The section passes through top of locules, showing inner (arrows) and outer (arrowhead) perianth whorls. Scale = 500 μm. (E) TS through fruit showing vascular bundles in fruit axis (arrows). Note inner perianth whorl seen adjacent to one carpel (arrowhead) and the sclerenchymatous nature of the fruit. Scale = 1 mm. UF19442-70248. (F) Longitudinal section through seed showing poorly preserved seed coat (black arrow) filled with abundant septate fungal hyphae (arrowhead). Note the thin pericarp (white arrow), which is only a few cell layers thick. Scale = 100 μm. CMNH slide 4305. (G) 3D reconstruction of a portion of infructescence from μCT data with a single vertical row of fruits and partial peduncle represented. Note free lobes of inner perianth whorl near top of fruit (arrowheads), stellate ridges on fruit (two of which sit lower), and shallow central depression best seen on lowermost fruit (arrow) and in reconstruction shown in (H). Scale = 1 mm. CMNH P3771. (H) 3D reconstruction of single fruit from specimen shown in (B) and (E), seen from above (top image) and laterally (middle and bottom images). Ridges on top of each carpel (black arrowheads) are higher and come to a point where they abut inner perianth elements, which are free from carpels near top of fruit (white arrow). Note how two ridges sit lower than others, and rim of tissue at base of fruit (white arrow) where outer perianth elements were attached. Some fusion of structures shown in 3D reconstructions, such as in perianth (middle image) are artifacts of segmentation process used to generate the 3D reconstructions. Scale = 1 mm. UF19442-70248. (I) Digital TS through infructescence showing three fruits attached at same node (arrowheads) and scattered vascular bundles in peduncle. Scale = 2 mm. CMNH P3771. (J) Two vascular bundles in peduncle showing preserved xylem (arrow), inner and outer fiber caps (arrowheads), and thin-walled cortical cells. Scale = 100 μm. CMNH slide 4239.

**Species**—***Viracarpon elongatum*** Sahni ex Chitaley & Patil emend. Matsunaga, S.Y. Smith, & Manchester.
*Basionym: Viracarpon elongatum* Chitaley & Patil, Botanique 2: 44–46. 1971.
*Synonyms: Shuklanthus superbum* Verma, Jour. Palaeon. Soc. India 3: 196. 1958.
*Viracarpon chitaleyi* Patil, Botanique 3: 25–26. 1972.
*Lectotype*: Specimen No. 3 Moh/P-5 (Institute of Science Nagpur), Chitaley & Patil, 1971, Plate 1, Fig. 1. Lectotype and other figured specimens have been lost.
*Neotype*: The neotype is designated here as specimen P3771 (CMNH; Fig. 5A, 5C, 5D, 5G and 5I).
*Additional material*: FLMNH UF19442-70248 10 (Figs. 5B, 5E and 5H).
*Type locality, stratigraphy, and age*: Mohgaonkalan, Deccan Intertrappean Beds, India, Late Maastrichtian.
*Other occurrences*: Takli, Marai Patan—Deccan Intertrappean Beds, India, Late Maastrichtian–early Danian.

*Emended specific diagnosis*—Infructescences slender, elongate, up to 6.5 cm long and 0.5 cm wide; peduncle of uniform width throughout, with two to three cycles of vascular bundles; fruits in whorls of three, up to 2.5 mm wide and 2.0 mm long; lateral walls of adjacent carpels in ovary free; distal surface of fruit with six shallow ridges; perianth in two whorls, outer perianth whorl free, inner whorl partially fused to ovary.

Description:

Infructescence structure: *V. elongatum* forms slender, elongate, spike-like infructescences up to 5.0 mm wide and 6.5 cm long, with fruits forming five to six orthostichies (Figs. 5A and 5B). Unlike *V. hexaspermum* the peduncle is not expanded where fruits are attached and is instead a uniform diameter throughout, ca. 1.5 mm. Fruits are sessile and organized into alternating whorls of three (Fig. 5I), although one specimen was originally described as having helical arrangement (Patil, 1972).

Fruit structure: Fruits are relatively small, measuring 2.0–2.2 mm in diameter and 1.3–1.5 mm tall, but exhibit a similar gynoecial morphology consisting of six single-seeded carpels fused centrally and with the lateral carpel walls free (Fig. 5E). Seeds, when preserved, have a thin seed coat, show no evidence of the distal lenticular structure seen in the seeds of *V. hexaspermum*, and are often colonized by septate fungal hyphae (Fig. 5F). Preservation is insufficient to determine seed coat thickness or histology. No projecting structures are seen above the ovary such as stylar spines or perianth elements (Fig. 5C). However, the upper surface of each fruit has six raised ridges positioned medially on top of each carpel, four of which sit slightly higher than the other two. A shallow depression is present in the center of the ridges, directly above the axis of the fruit (Fig. 5G and 5H). The pericarp is thin, approximately two to three cell layers thick where it can be distinguished from the inner perianth, and sclerenchymatous (Figs. 5E and 5F). No clear evidence of germination sutures or dehiscence structures have been observed.

Vasculature: The peduncle contains usually two to three cycles of vascular bundles arranged irregularly in the cortex, which is parenchymatous (Figs. 5I and 5J). Vascular bundles are collateral, with fibers forming caps to the inside and outside of the bundle, or sometimes surrounding the bundle completely. Xylem tracheary elements are preserved in some specimens, consisting of ∼8–16 cells measuring 10–25 μm in diameter. Phloem is not preserved (Fig. 5J). Each fruit is vascularized by six bundles that run through the central axis and curve over the top of the locules, corresponding to each ridge on the surface of the fruit (Fig. 5E). However, it is not clear whether the bundle vascularizes the seed as it does in *V. hexaspermum* and *Viracarpon phytolaccoides.*

Perianth: *V. elongatum* possesses an outer perianth whorl of six elements, free from and arranged opposite each carpel (Figs. 5C and 5D). Additionally, at the distal end of each carpel is an outer layer of sclerenchymatous tissue that is free from the ovary and the ridges on top of each fruit (Figs. 5C–5H). This layer may be derived from an inner perianth whorl partially fused to the ovary wall like that of *V. hexaspermum*, as has been suggested by previous investigators (Patil, 1972).

**Figure 6:**
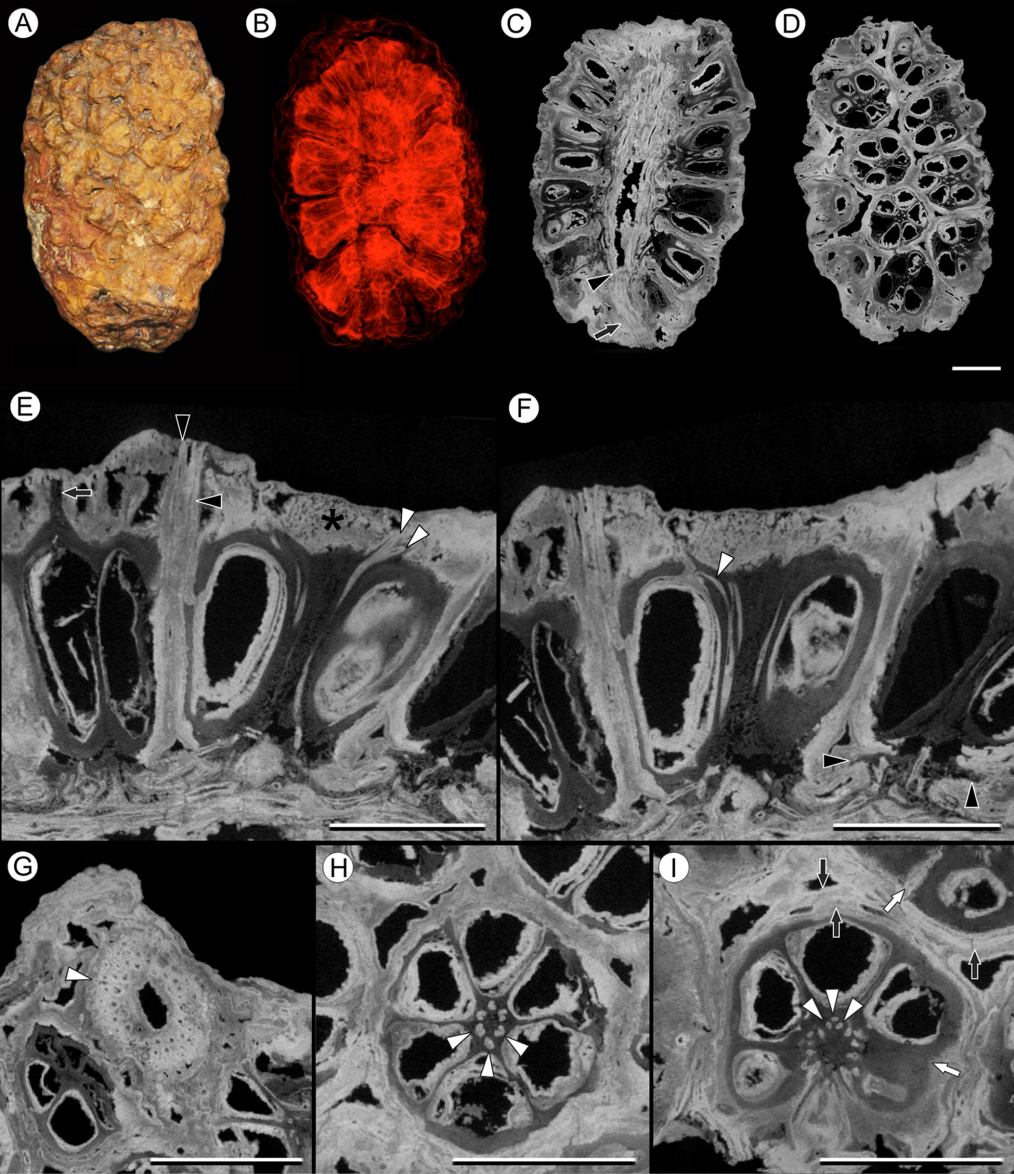
*Viracarpon* (syn. *Coahuilocarpon*) *phytolaccoides* (Cevallos-Ferriz, Estrada-Ruiz & Perez-Hernandez) Matsunaga, S.Y. Smith, & Manchester comb. nov. (A) External view of specimen shown in (B–I). (B) Volume rendering of infructescence illustrating 3D helical arrangement of fruits forming clear orthostichies. (C) Median digital longitudinal section (DLS) through infructescence. Note that base of peduncle (arrow) is much narrower than in region where fruits are attached (transition indicated by arrowhead). (D) Digital tangential section through infructescence, showing arrangement of the fruits. Note similarity with Fig. 1G, which depicts a similar section of a *V. hexaspermum* infructescence. (E) DLS through a fruit showing trajectory of two of vascular bundles (white arrowheads) that continue to edge of rock matrix. Note contrast between darker inner tissues of fruit and lighter distal tissues (asterisk). Darker tissue forms a projection extending through lighter tissues to edge of the rock matrix (arrow), seen in the fruit on the far left; layers probably representing perianth are indicated by black arrowheads. (F) DLS through a fruit showing trajectory of third vascular bundle, which can be seen arcing into opening at the top of locule (white arrowhead). Also note flanges of base of fruit on far right (black arrowheads), which may represent remnants of an outer perianth whorl; these flanges can also be seen in some fruits in (C). (G) Digital transverse section (DTS) through peduncle showing scattered arrangement of vascular bundles and smaller outermost bundles (white arrowhead). (H) DTS through a fruit showing six locules arranged around central axis with six vascular bundles (arrowheads). (I) DTS through a fruit taken toward the top of the locules, showing trifurcation of vascular bundles (arrowheads). Some of outer layers of fruit may represent perianth elements (black arrows). Note the gaps and indentations between adjacent carpels, indicating carpels were free at least in distal regions (write arrows). All scale bars = 5 mm. IGM-PB 9884.

**Species**—***Viracarpon phytolaccoides*** (Cevallos-Ferriz, Estrada-Ruiz & Perez-Hernandez) Matsunaga, S.Y. Smith, & Manchester comb. nov.
*Basionym: Coahuilocarpon phytolaccoides* Cevallos-Ferriz, Estrada-Ruiz & Perez-Hernandez, Am. J. Bot. 95: 79. 2008.
*Holotype*: IGM-PB 1244 (Universidad Nacional Autonoma de Mexico).
*Other specimens studied*: One additional specimen was studied by μCT (IGM-PB 9884, Fig. 6).
*Stratigraphy and age*: Cerro del Pueblo Formation, late Campanian (72.3–71.3 Ma).
*Emended specific diagnosis*—Infructescences up to 4.5 cm long and 2.5 cm wide, with helically arranged fruits; peduncle wider in region of fruit attachment, with scattered vascular bundles in several cycles; outer vascular bundles smaller than innermost ones; fruits up to 7.0 mm wide and 8.0 mm long; vascular bundles of fruit trifurcating above locule, one bundle entering seed, two bundles entering style.

Description:

Infructescence structure: *V. phytolaccoides* forms compact infructescences, 2.0–4.4 cm long by 1.5–2.3 cm wide (Figs. 6A–6D; Cevallos-Ferriz, Estrada-Ruiz & Perez-Hernandez, 2008), oblong to ellipsoidal in shape, and bearing helically arranged fruits in usually eight orthostichies (Fig. 6D). The infructescence axis is approximately 6 mm in diameter where fruits are attached and tapers to ∼2.5 mm at the proximal end, just below fruit attachment (Fig. 6C).

Fruit structure: Fruits of *V. phytolaccoides* are up to 7.5 mm wide distally, narrowing slightly toward the base, and 7.0–8.0 mm tall (Fig. 6E and 6F). Measurements are based on the most complete and uncompressed specimens, which tended to be among the largest in the size spectrum. Many smaller specimens were too compressed to yield reliable measurements, but may have smaller fruits. The gynoecium comprises six carpels, each containing a single-seeded locule, which are arranged around a central axis (Figs. 6D–6I). Ovary fusion is somewhat ambiguous, but they appear to be completely fused for most of their length and free distally, as indicated by gaps and indentations between adjacent carpels (Fig. 6I). However, the preservation makes it difficult to determine unequivocally whether proximal regions of the fruit are truly fused or just very closely appressed. In μCT sections the pericarp is composed of an inner darker and less dense region, and an outer denser region on the distal surface of the fruit (Figs. 6E and 6F). These density differences may reflect histological differences between two tissues of the pericarp. Cevallos-Ferriz, Estrada-Ruiz & Perez-Hernandez (2008) documented isodiametric cells in the pericarp, but preservation was too incomplete overall to determine other details of pericarp anatomy. Above each locule, the darker inner tissue forms a thin tapering structure that projects through the lighter outer tissue layer (Fig. 6E, arrow). While its position suggests the structure is probably the base of a style, available specimens do not retain any of the rock matrix surrounding the infructescence in which projecting styles or perianth would be preserved. Germination sutures and dehiscence structures were not observed.

Vasculature: Vascular bundles in the infructescence axis are often poorly preserved, but some specimens show three or four cycles of vascular bundles present in the cortex, surrounding a narrow central pith (Fig. 6G). The outermost vascular bundles appear smaller than the inner ones (Fig. 6G). Individual fruits are vascularized by six bundles that run through the axis of the fruit, opposite the carpels (Fig. 6H). At the distal end of the fruit, near the top of the locules, each vascular bundle trifurcates (Fig. 6I, arrowheads): the middle strand enters the distal end of the locule wall (Fig. 6F), and the other two strands extend over the top of the locule and out of the rock matrix (Fig. 6E). The vascularization of the locule is characteristic of apical placentation of ovules within the locule, consistent with the observations of Cevallos-Ferriz, Estrada-Ruiz & Perez-Hernandez (2008).

Perianth: Perianth elements in *V. phytolaccoides* specimens are poorly preserved, making it difficult to resolve the precise number and organization relative to the carpels. Transverse sections show an outer tissue layer that may or may not be fused to the pericarp (Fig. 6I), which can sometimes be seen in longitudinal sections extending all the way to the edge of the rock specimen (Fig. 6E). In longitudinal sections, small flanges can be seen at the base of the fruit below the ovary (Figs. 6C and 6F), possibly corresponding to an outer perianth whorl.

## Discussion

### Two species of *Viracarpon* in India

Four species of *Viracarpon* have been formally erected based on specimens collected from the Deccan Intertrappean Beds of India: *V. hexaspermum*, *V. sahnii*, *V. elongatum*, and *V. chitaleyi* (Table 1; Sahni, 1934, 1944; Chitaley, Shallom & Mehta, 1969; Patil, 1972). Previous researchers proposed synonymizing *V. hexaspermum* and *V. sahnii* (Nambudiri & Tidwell, 1978), as well as *V. elongatum* and *V. chitaleyi* (Bande & Awasthi, 1986). Additionally, specimens originally interpreted as inflorescences and described under the name *Shuklanthus superbum* (Verma, 1958) were formally synonymized with *V. elongatum* (Chitaley & Patil, 1971). Our observations, based on the re-examination of fruit morphology undertaken here, support these conclusions and indicate that there are only two recognizable species of *Viracarpon* in the Deccan Intertrappean Beds: the more globose infructescences of *V. hexaspermum* and the narrower, elongate infructescences of *V. elongatum*.

**Table 1:** Morphological comparison of *Viracarpon* species.

Original taxonomy	Revised taxonomy	Infructescence shape	Infructescence width	Max fruit width (mm)	Perianth	Reference
***V. hexaspermum***	*V. hexaspermum*	Globose—oblong	Up to 20 mm	6	One whorl observed	Chitaley (1954); This study
***V. sahnii***	*V. hexaspermum*	Globose—oblong	Up to 25 mm	6	Not mentioned	Chitaley, Shallom & Mehta (1969)
***V. elongatum***	*V. elongatum*	Slender, elongate	Up to 5 mm	2.2	Two whorls	This study
***S. superbum***	*V. elongatum*	Slender, elongate	Up to 5.2 mm	2.7	Two whorls	Verma (1958)
***V. chitaleyi***	*V. elongatum*	Slender, elongate	Not mentioned	2	Two whorls	Patil (1972)
***C. phytolaccoides***	*V. hexaspermum*	Globose—oblong	Up to 23 mm	7.5	One whorl observed	Cevallos-Ferriz, Estrada-Ruiz & Perez-Hernandez (2008); This study

**Notes:**

Morphological comparison of *Viracarpon* species described from the Deccan Intertrappean Beds of India (including *Shuklanthus superbum*) and *Viracarpon* (=*Coahuilocarpon) phytolaccoides* from Mexico. Note the morphological similarities between *V. hexaspermum* and *V. sahnii*, and between *V. elongatum, V. chitaleyi*, and *S. superbum*, supporting their synonymy.

*Viracarpon sahnii*, now synonymized with *V. hexaspermum*, was distinguished based on the absence of trichomes on the fruits and the presence of a structure described as a “hump” above each locule (Chitaley, Shallom & Mehta, 1969). However, our observations based on multiple μCT scans and 3D reconstructions of *V. hexaspermum* indicate that the “hump” described by Chitaley, Shallom & Mehta (1969) corresponds to the ridges on the distal ends of fruits formed by the persistent styles. These ridges, as well as the spine-like projections of styles and perianth, are the source of most of the ambiguity and disagreements on the morphology of *V. hexaspermum* because the longitudinal profile of the fruit can look very different depending on the plane of section. Moreover, the curvature and relief of these ridges appears to be somewhat variable within the species, as seen when comparing 3D reconstructions of two different fruits, and may therefore appear more prominent in some specimens than others. Therefore, we formally synonymize these two names into *V. hexaspermum*, which has priority. Interestingly, our 3D reconstructions (Figs. 3A–3E) most closely resemble that of Chitaley (1954), which was the first published reconstruction of *V. hexaspermum*; this reconstruction was considered inaccurate by other researchers, who proposed alternative reconstructions (Nambudiri & Tidwell, 1978; Bande & Awasthi, 1986). Those alternative reconstructions are not consistent with our μCT scan imagery. Regarding the absence of trichomes on the fruits, this may reflect either preservational differences (Nambudiri & Tidwell, 1978) or variation within the species.

The more elongate, slender infructescences of *V. elongatum* were named by Sahni (1944) and subsequently validated with designation of lectotype by Chitaley & Patil (1971). Similar fossils originally interpreted as inflorescences were described by Verma (1958) as *S. superbum*. Verma (1958) noted their affinities with *V. hexaspermum*, but at the time no specimens of *V. elongatum* were available for comparison and no detailed description of the species existed. Specimens similar to *Shuklanthus* were later collected and described by Chitaley & Patil (1971), who recognized their similarity with *V. elongatum* and designated *Shuklanthus superbum* as a junior synonym of *V. elongatum*. Specimens of *V. elongatum* examined in this study are indistinguishable from the published descriptions and figures of *Shuklanthus*, and support the concept of synonymy for the two species (Table 1). *V. chitaleyi* was segregated by Patil (1972) who indicated that it differs from *V. elongatum* in the number of orthostichies seen on infructescences, and in the inferred arrangement of fruits along the peduncle (helical vs. whorled). However, as recognized by Bande & Awasthi (1986), these differences probably represent normal variation within a species (number of orthostichies is also somewhat variable in *V. hexaspermum*) and therefore *V. chitaleyi* should be formally merged with *V. elongatum*, which has priority.

Despite notable differences in infructescence morphology, fundamental floral characters are shared between *V. hexaspermum* and *V. elongatum* that support their inclusion in the same genus. These include a gynoecium comprised of six single-seeded carpels fused centrally and with the lateral carpel walls free, fruits vascularized by six bundles that each correspond to one carpel, and a perianth consisting of at least one whorl of six elements arranged opposite the carpels. Additionally, both species have a similar vascular anatomy within the peduncle, and exhibit a stellate pattern of ridges on the distal surface of the fruits. In *V. hexaspermum* these ridges are formed by the bases of six unfused styles, which form spine-like projections that are persistent in mature fruits. The similarities in fruit vascularization and the stellate pattern of ridges in *V. elongatum* suggests that it probably had a semicarpous morphology like that of *V. hexaspermum*, in which the ovaries are partially fused and the styles are free. However, the ridges are less prominent than those seen in *V. hexaspermum*, and do not form long projections in the mature fruit. The consistent absence of projecting structures in all documented specimens of *V. elongatum* indicates that this is unlikely to be a product of taphonomy (abrasion or degradation) and may indicate that unlike *V. hexaspermum*, *V. elongatum* did not retain its styles at maturity.

### Alternative morphological interpretations

We considered some alternative morphological interpretations of *Viracarpon*, including whether fruits were schizocarpous at maturity, if the carpels were truly semicarpous or were instead apocarpous as in *Illicium* L., and whether *V. hexaspermum* and *V. elongatum* were different developmental stages of the same species:

First, we consider it unlikely that the ovary of *Viracarpon* was completely fused, forming a schizocarpous fruit at maturity, rather than fused centrally with free lateral walls. Nearly all specimens of *Viracarpon* are whole infructescences, and dispersed fruits are almost always intact. Moreover, all specimens studied exhibit central fusion for most of their length, with little evidence that they separated into mericarps. While some planes of section show complete separation of adjacent carpels in *V. elongatum* (Fig 5D), this only appears to be present in very distal regions of some fruits. These features, along with the absence of dehiscence structures on the fruits, suggest that the fruits were probably indehiscent and dispersed as a unit rather than as separate mericarps. However, more data are needed to confirm fruit type and dispersal mode.

Second, in extant *Illicium* the gynoecium is apocarpous, with carpels attached laterally to a central columnar receptacle (Romanov, Bobrov & Endress, 2013). This morphology is somewhat reminiscent of *Viracarpon*, and raises the question of whether the gynoecium was truly semicarpous or was instead apocarpous, with carpels attached to a receptacle rather than centrally connate. This question comes down to whether the central axis of *Viracarpon* was composed of tissues of the carpels or the floral receptacle. The lack of specimens preserved in much earlier stages of development makes it difficult to resolve this question unequivocally. However, the continuity of the central axis and styles in *V. hexaspermum* supports carpel homology of the central axis; this does not appear to be the case in *Illicium*, in which the receptacle and carpels are readily distinguishable. Information on the developmental morphology of *Viracarpon*, as well as future comparisons with *Illicium* or other taxa with similar gynoecial morphology will hopefully clarify this. Nevertheless, while the carpels of *Viracarpon* are partially fused, they are very nearly apocarpous. This is potentially relevant to understanding the evolution of carpel fusion in monocots, and would be interesting to explore further in the context of *Viracarpon*’s systematic affinities, if they can be resolved.

Finally, regarding whether *V. elongatum* and *V. hexaspermum* might represent different developmental stages of the same plant, this also seems unlikely because both appear to be in mature fruiting condition. Major differences between the two species include the overall size of fruits, the structure of the infructescences, which are nearly head-like in *V. hexaspermum* and more spike-like in *V. elongatum*, the number of orthostichies on infructescences, and number of vascular bundles in the peduncle. Such a developmental transition would require significant changes in the number and arrangement of fruits, an increase in the number of vascular bundles in the peduncle, and an expansion in the diameter of the peduncle. Moreover, although some specimens of *V. elongatum* were originally described as flowers (Verma, 1958), the size of the locules, lack of floral structures like styles or stamens, and the presence of a seed coat in some specimens (Fig. 5F) indicates that fruits of *V. elongatum* were mature or close to maturity and do not represent an early developmental stage.

### Reassignment of *C. phytolaccoides* from the Campanian of Mexico to *Viracarpon*

*Coahuilocarpon phytolaccoides* from the Campanian of Mexico, was originally placed in the Phytolaccaceae based on its resemblance to members of the extant genus *Phytolacca* (Cevallos-Ferriz, Estrada-Ruiz & Perez-Hernandez, 2008). Vascular anatomy of the peduncles was poorly preserved in the type material, and affinities with monocots were not considered by the original authors. Most of the morphological interpretations of Cevallos-Ferriz, Estrada-Ruiz & Perez-Hernandez (2008) are consistent with our observations of *Coahuilocarpon* specimens aided by μCT investigations. However, we were unable to document any characters to support the interpretation of the ovules as campylotropous either in our μCT data or the original publication. Furthermore, we consider the campylotropous state to be unlikely because nearly all monocots with apical placentation have orthotropous ovules (Dahlgren, Clifford & Yeo, 1985).

Based on our μCT studies of *Coahuilocarpon*, our observations of *Phytolacca*, and comparisons with *Viracarpon*, we conclude that *Coahuilocarpon* is much more like *Viracarpon*, particularly *V. hexaspermum*, than *Phytolacca* and other extant genera. Similarities with *V. hexaspermum* include compact infructescences bearing sessile, helically arranged fruits falling within the size range of *V. hexaspermum*, a peduncle with scattered vascular bundles that is wider in the zone where fruits are attached, vascular bundles that are larger toward the center of the peduncle, fruits comprised of six carpels and a central axis around which the locules are arranged, six vascular bundles that run through the central axis of the fruit and into a distal structure (the style in *Viracarpon*; unknown in *Coahuilocarpon*), and apical placentation. By contrast, fruits of *Phytolacca* are consistently pedicellate, vary in carpel number, and exhibit basal placentation. Moreover, the presence of six carpels and scattered vascular bundles in the peduncle of *Coahuilocarpon*, revealed in μCT scans, indicates monocot affinities. Taken together, this suite of morphological similarities suggests *C. phytolaccoides* is very closely related to *V. hexaspermum*, and leads us to conclude that they represent the same genus. Owing to some differences and uncertainties in morphology, they are best treated as separate species within *Viracarpon*. Accordingly, we consider *Coahuilocarpon* to be a junior synonym of *Viracarpon*, and suggest the new combination, *V. phytolaccoides* (Cevallos-Ferriz, Estrada-Ruiz & Perez-Hernandez) Matsunaga, S.Y. Smith, & Manchester comb. nov. Differences distinguishing *V. phytolaccoides* from *V. hexaspermum* include lateral fusion of carpels, lack of information on distal structures of the fruit such as stylar spines or trichomes owing to mode of preservation, and uncertainties in the perianth morphology. Perianth morphology is also an area of uncertainty for *V. hexaspermum* and *V. elongatum* (Table 1); whereas only one whorl is seen in fruits *V. hexaspermum*, there are probably two in *V. elongatum* and these observed differences could be developmental.

It would be helpful to know if *V. phytolaccoides* possessed stylar spines in mature fruits, but no documented specimens of *V. phytolaccoides* are embedded in an external rock matrix. In specimens of *V. hexaspermum*, the persistent styles and perianth spines are always preserved in the surrounding matrix, and are not seen in specimens where fruits are exposed on the external surface of the rock. These preservational features observed in the Indian *V. hexaspermum* suggest that it is possible such structures were present in the Mexican species, but are not seen in any of the specimens owing to the mode of preservation. Nevertheless, in fruits of *V. phytolaccoides*, the trajectory of the vascular bundles and the distal tapering of the pericarp in each carpel (Fig. 6E) together indicate that there was an attached structure above each locule at some point in development, probably a style. This suggests that the gynoecium of *V. phytolaccoides* was semicarpous like the Indian *Viracarpon* species, with a partially fused ovary and free styles. However, recovery of better preserved specimens will be necessary to determine whether stylar spines were present at maturity, and to resolve some of the other uncertainties in the morphology of *V. phytolaccoides*, such as details of the perianth and whether the ovaries are fully or partially fused proximally.

One notable feature of the vasculature in *V. phytolaccoides* fruits is the bifurcation of the vascular strand distal to where it branches to supply the seed. In *V. hexaspermum*, resolution in μCT scans is not sufficient to tell unequivocally whether this character is also present. However, it is possible the vascular strand entering the spine may branch to form two closely spaced smaller strands that run parallel to one another, as it sometimes appears in transverse sections through the spines (Figs. 4E and 4K).

### Biogeographic implications

The early geographic spread and evolutionary radiation of angiosperms coincides with a dynamic interval in India’s tectonic history. Following the initial breakup of Gondwana, at about 130 Ma India and Madagascar began rifting northward, separating from one another at about 90 Ma (Ali & Aitchison, 2008; Chatterjee, Goswami & Scotese, 2013; Gibbons, Whittaker & Müller, 2013). India subsequently spent millions of years in geographic isolation until its collision with Eurasia 40–50 Ma, with the precise timing of continental collision remaining uncertain and controversial (Ali & Aitchison, 2008; White & Lister, 2012; Bouilhol et al., 2013; Chatterjee, Goswami & Scotese, 2013; Jagoutz et al., 2015; Matthews, Müller & Sandwell, 2016). Although the extent of India’s biotic isolation is debated (Ali & Aitchison, 2008; Chatterjee & Scotese, 2010), the paleogeography of India should to some extent be reflected in the biogeographic relationships of the Deccan flora, with some elements representing endemic taxa and others exhibiting Gondwanan or Asian affinities. Such patterns have been documented for other components of India’s Cretaceous and Eocene biotas, including strong endemism of freshwater ostracod faunas (Whatley & Bajpai, 2006; Sharma, Bajpai & Singh, 2008), and both Laurasian and Gondwanan affinities for an array of animal taxa (Bajpai, 2009).

Owing to the unusual morphology of *Viracarpon* and challenges associated with resolving its taxonomic relationships, we speculated that it could represent a genus endemic to India. However, the broad geographic disjunction between Indian species of *Viracarpon* and *V. phytolaccoides* in Mexico suggests that *Viracarpon* was more widespread than previously known and may be present in other Cretaceous floras. This is broadly consistent with Maastrichtian and Eocene faunal data that indicate cosmopolitan relationships of many Indian taxa, particularly among vertebrates, with comparatively little evidence of endemism (Bajpai, 2009; Chatterjee & Scotese, 2010). It will be interesting to learn whether there are additional occurrences of *Viracarpon* in other Cretaceous assemblages, perhaps described under different names. Our examination of numerous museum specimens exhibiting a broad spectrum of preservation revealed that, depending on the mode of preservation, specimens of *Viracarpon* can look very different from one another, and without careful study or the right search image they are easily misidentified. As an example, specimens of *V. hexaspermum* were found at two different museums listed as “unidentified conifer.” Moreover, if preserved as compression or impression fossils rather than as 3D permineralizations, diagnostic characters would be difficult or impossible to recognize.

### Systematic affinities of *Viracarpon*

Affinities with several families have been suggested for *Viracarpon*, including the Araceae (Chitaley, 1954), Cyclanthaceae (Sahni, 1944), Pandanaceae (Nambudiri & Tidwell, 1978), and Scheuchzeriaceae (Verma, 1958) based primarily on infructescence morphology (Table 2). Some fundamental characters of *Viracarpon* that are potentially taxonomically informative include a semicarpous gynoecium composed consistently of six carpels, six-locular fruits, a single seed per locule, and apical placentation. This combination of characters is not seen in any taxa within the families previously proposed as having affinities with *Viracarpon* (Table 2). The greatest morphological similarity is with the Araceae, but all members have fully fused carpels and few genera have six-locular fruits; only two genera have non-berry fruits (Mayo, Bogner & Boyce, 1997, 1998). With respect to other potential relationships within monocots, apical placentation is seen in some members of Commelinaceae, Mayaceae, Poaceae, Eriocaulaceae, Alismatales (Araceae, Potamogetonaceae, Ruppiaceae, Posidoniaceae, Cymodoceaceae, and Zosteraceae), and Acoraceae (Dahlgren, Clifford & Yeo, 1985; Igersheim, Buzgo & Endress, 2001). Of these taxa, to our knowledge only the Alismatales contains members with six carpels, and many have partially fused carpels (Igersheim, Buzgo & Endress, 2001). However, *Viracarpon* does not clearly or easily fit into any modern family within Alismatales, particularly when some of its more unusual morphological characters are considered, such as the presence of stylar and perianth spines, adnation of the perianth to the gynoecium, and the densely trichomatous nature of *V. hexaspermum* fruits. It is therefore possible that it represents an extinct family within Alismatales.

**Table 2:** Morphological comparison of *Viracarpon* and extant monocots.

Character	*Viracarpon*	Araceae	Pandanaceae	Scheuchzeriaceae	Cyclanthaceae
**Gynoecium fusion**	Semicarpous (ovary fused, styles free)	Syncarpous	Apocarpous–syncarpous	Apocarpous or basally connate	Syncarpous (styles free or fused)
**Carpel number**	6	1–8	1–Many	3 or 6	4
**Locule number**	6	1–Many	1–Many	3 or 6	1
**Seeds per locule**	1	1–Many	1–Several	1–2	Many
**Placentation**	Apical	Variable (basal–apical, axile–parietal)	Marginal, axile, or basal	Basal or axile	Parietal or apical
**Perianth elements**	6 (inner perianth)	4, 6, 8, or absent	3–4 or absent	6	4 or absent
**Inflorescence type**	Spike, head	Spike (spadix)	Panicle, raceme, head, or spike	Raceme	Spike
**References**	This study	Dahlgren, Clifford & Yeo (1985); Mayo, Bogner & Boyce (1997, 1998)	Dahlgren, Clifford & Yeo (1985); Watson & Dallwitz (1992 onwards); Stone, Huynh & Poppendieck (1998)	Dahlgren, Clifford & Yeo (1985); Watson & Dallwitz (1992 onwards); Haynes, Les & Holm-Nielsen (1998)	Dahlgren, Clifford & Yeo (1985); Watson & Dallwitz (1992 onwards)

**Notes:**

Comparison of genus *Viracarpon* with Araceae, Pandanaceae, Scheuchzeriaceae, and Cyclanthaceae, which were proposed as having affinities with *Viracarpon* by previous researchers. *Viracarpon* exhibits the greatest amount of similarity with Araceae, but differs from all Araceae in having a semicarpous gynoecium with free styles.

*Viracarpon* was present during a period of diversification among monocot angiosperms and exhibits a combination of characters that is reminiscent of, but not present in any, extant alismatalean families. Extinction prunes lineages over time, erasing the history of clades (Marshall, 2017), and therefore character combinations not present in extant taxa are expected in the fossil record. Considering this, the possibility that *Viracarpon* belongs to the stem or crown-group of a modern family cannot be completely discounted and should be further explored. Because the morphological characters of *Viracarpon* in themselves provide no clear answers to its taxonomic affinities, phylogenetic analyses will be essential for resolving its systematic placement among monocots as either a member of an extinct family, or as an extinct genus of a modern family. Such analyses would also provide a framework for addressing broader questions on monocot evolution. Owing to the scale of such an analysis, and the fact that we still lack the level of detail in extant monocot anatomy and morphology that would be necessary to accomplish this, it will be the subject of a future study.

The additional characters documented by this study will be essential for including *Viracarpon* in future phylogenetic analyses. Three-dimensional reconstructions based on μCT scans provided a valuable means of resolving its complex external morphology, and digital sectioning of specimens was necessary for confirming the homology of the spines and visualizing other important characters such as the vascular architecture within fruits. This demonstrates that μCT remains a viable and powerful tool for paleobotanical research, especially for non-destructive study of museum specimens.

Although the systematic affinities of *Viracarpon* remain unresolved, its widespread distribution provides additional evidence indicating that monocots were broadly distributed and their radiation well under way by the end of the Cretaceous. This is congruent with the appearance of relatively derived monocot clades during the Late Cretaceous including fossils of Triuridaceae (Turonian; Gandolfo, Nixon & Crepet, 2002), Arecaceae (Santonian; Berry, 1914), Zingiberales (Campanian; Friis, 1988), and Poaceae (Maastrichtian; Prasad et al., 2011). Among these taxa only the Arecaceae exhibit broad geographic distributions in the fossil record by the Maastrichtian (Ancibor, 1995; Gee, 2001; El-Soughier et al., 2011; Manchester et al., 2016). The first appearance of monocots in the Barremian–Aptian (∼125 Ma; Friis, Pedersen & Crane, 2004; Doyle, Endress & Upchurch, 2008; Iles et al., 2015), their sparse early fossil record, and the appearance of several derived lineages by the Maastrichtian together imply a largely invisible radiation of monocots during much of the Cretaceous (Crepet, 2008). The unusual morphology of *Viracarpon* and occurrences of other similarly enigmatic monocots in the Deccan Intertrappean Beds (e.g., *Tricoccites*; Chitaley, 1956; Bonde, 1985) raise the possibility that this Cretaceous diversification of monocots may be characterized by widespread extinct or stem taxa. Resolving the systematic relationships of *Viracarpon* in future phylogenetic analyses and continued study of the Deccan monocots will hopefully shed light on these questions and lead to a better understanding of monocot diversification, distribution, and morphological evolution.

## Conclusion

Fossils of *Viracarpon* were first collected over a century ago by Hislop (1853). Since then, understanding the morphology of this genus, particularly *V. hexaspermum*, has been challenging and its taxonomic affinities remain elusive. In this study we undertook a morphological reinvestigation of *Viracarpon* by examining type material and studying museum specimens using X-ray μCT. Three-dimensional reconstructions of the fruits and visualization of specimens in multiple planes of section allowed us to clarify conflicting interpretations of fruit morphology and identify additional characters. These characters will be useful in refining the potential systematic affinities in future phylogenetic analyses. Comparisons of our reconstructions and observations with those of previous researchers revealed that the reconstruction of Chitaley (1954), the first to be published, was accurate with respect to the overall morphology of the fruits, capturing aspects of the complex external morphology resolved here using μCT. Further, we tested the hypothesis that *Viracarpon* was endemic to India by comparing it to *C. phytolaccoides* from the late Campanian of Mexico. Morphological similarities and the presence of diagnostic characters of *Viracarpon* indicate that *Coahuilocarpon* is a junior synonym of *Viracarpon*, leading to the recognition of *V. phytolaccoides* comb. nov. The geographic disjunction between these two occurrences presents an intriguing biogeographic conundrum and indicates that, rather than being endemic to India, the genus was once widespread. *Viracarpon* provides additional evidence for characterizing the largely invisible Cretaceous radiation of monocots. Its future inclusion in phylogenetic analyses will hopefully aid in resolving its systematic placement and elucidate evolutionary patterns and processes underlying the diversification of monocot angiosperms.

## Supplemental Information

10.7717/peerj.4580/supp-1 Supplemental Information 1 3D reconstruction shown in text Figs. 3D&3E. File must be opened in Adobe Acrobat Reader. 3D model can be freely rotated and sectioned in custom planes. Note that this is a surface rendering and does not show internal details of the specimen. Perianth is omitted from this reconstruction. Based on specimen NHMUK V35059.

10.7717/peerj.4580/supp-2 Supplemental Information 2 Serial digital transverse sections through top of fruit in text Fig. 1E, showing channels in fruit, perianth, and persistent styles. NHMUK V35059.

10.7717/peerj.4580/supp-3 Supplemental Information 3 Corresponding video for text Fig. 4G. Serial digital transverse sections through fruit showing vascular bundle that enters the top of the locule and continues into style. NHMUK V35059.

10.7717/peerj.4580/supp-4 Supplemental Information 4 Serial digital transverse sections through entire infructescence from NHMUK specimen V35059.

10.7717/peerj.4580/supp-5 Supplemental Information 5 Surface rendering of specimen NHMUK V35036, shown in text Fig. 2A&2B, in which infructescence is preserved as a three-dimensional impression.

10.7717/peerj.4580/supp-6 Supplemental Information 6 Serial digital transverse sections through entire specimen NHMUK V35036. The infructescence is preserved primarily as a three-dimensional impression in the chert. However, some tissues like those of the perianth are permineralized in the rock matrix.

10.7717/peerj.4580/supp-7 Supplemental Information 7 Volume rendering of specimen NHUMUK V35036 showing overall shape of infructescence. Note the compact, nearly globose shape of this particular infructescence, and the fuzzy appearance produced by style and perianth spines.

## Additional Information and Declarations

### Data Availability

The following information was supplied regarding data availability: μCT data for NHMUK specimens are archived by the museum Imaging and Analysis Centre (IAC), and can be accessed by contacting IAC staff. http://www.nhm.ac.uk/our-science/departments-and-staff/core-research-labs/imaging-and-analysis-centre.html. μCT data for all other specimens are archived on MorphoSource (https://www.morphosource.org/Detail/ProjectDetail/Show/project_id/430) under project P430 (title: Viracarpon, an enigmatic monocot from the Late Cretaceous).
