# Correction: Reinvestigating an enigmatic Late Cretaceous monocot: morphology, taxonomy, and biogeography of *Viracarpon*

**DOI:** 10.7717/peerj.4580/correction-1

**Published:** 2018-11-30

**Authors:** Kelly K.S. Matsunaga, Selena Y. Smith, Steven R. Manchester, Dashrath Kapgate, Deepak Ramteke, Amin Garbout, Herminso Villarraga-Gómez

**Affiliations:** 1 Earth and Environmental Sciences, University of Michigan, Ann Arbor, MI, USA; 2 Florida Museum of Natural History, University of Florida, Gainesville, FL, USA; 3 Department of Botany, Jashbhai Maganbhai Patel College, Bhandara, Maharashtra, India; 4 Imaging and Analysis Centre, Natural History Museum London, London, UK; 5 Nikon Metrology, Inc., Brighton, MI, USA

Corrigendum:

After publication, the authors noticed that the fossil species *Coahuilacarpon phytolaccoides* was misspelled. It was originally written as *Coahuilocarpon*, with an "o" and not an "a". This error has been amended throughout the article.

