# Supplementary figures and images for: Reinvestigating an enigmatic Late Cretaceous monocot: morphology, taxonomy, and biogeography of Viracarpon
Source: PeerJ. 2018 Apr 6;6:e4580. doi: 10.7717/peerj.4580 (PMC5890723; doi:10.7717/peerj.4580)

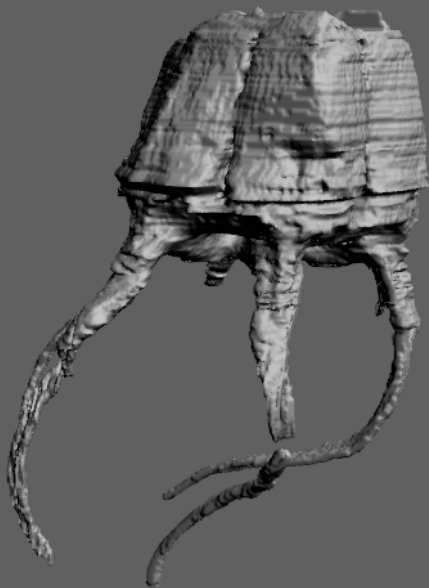

Supplement: Supplemental Information 1 — File must be opened in Adobe Acrobat Reader. 3D model can be freely rotated and sectioned in custom planes. Note that this is a surface rendering and does not show internal details of the specimen. Perianth is omitted from this reconstruction. Based on specimen NHMUK V35059. [file peerj-06-4580-s001.pdf]
